# Altered gray matter structural covariance networks in drug-naïve and treated early HIV-infected individuals

**DOI:** 10.3389/fneur.2022.869871

**Published:** 2022-09-20

**Authors:** Ruili Li, Yuxun Gao, Wei Wang, Zengxin Jiao, Bo Rao, Guangxue Liu, Hongjun Li

**Affiliations:** ^1^Department of Radiology and Nuclear Medicine, Xuanwu Hospital, Capital Medical University, Beijing, China; ^2^Department of Radiology, Beijing Youan Hospital, Capital Medical University, Beijing, China; ^3^Beijing Key Laboratory of Magnetic Resonance Imaging and Brain Informatics, Beijing, China; ^4^Department of Radiology, Zhongnan Hospital of Wuhan University, Wuhan, Hubei, China; ^5^Department of Natural Medicines, School of Pharmaceutical Sciences, Peking University Health Science Center, Beijing, China

**Keywords:** HIV, cognitive function, combination antiretroviral therapy, 3D-T1WI, structural covariance network

## Abstract

**Background:**

While regional brain structure and function alterations in HIV-infected individuals have been reported, knowledge about the topological organization in gray matter networks is limited. This research aims to investigate the effects of early HIV infection and combination antiretroviral therapy (cART) on gray matter structural covariance networks (SCNs) by employing graph theoretical analysis.

**Methods:**

Sixty-five adult HIV+ individuals (25–50 years old), including 34 with cART (HIV+/cART+) and 31 medication-naïve (HIV+/cART–), and 35 demographically matched healthy controls (HCs) underwent high-resolution T_1_-weighted images. A sliding-window method was employed to create “age bins,” and SCNs (based on cortical thickness) were constructed for each bin by calculating Pearson's correlation coefficients. The group differences of network indices, including the mean nodal path length (Nlp), betweenness centrality (Bc), number of modules, modularity, global efficiency, local efficiency, and small-worldness, were evaluated by ANOVA and *post-hoc* tests employing the network-based statistics method.

**Results:**

Relative to HCs, less efficiency in terms of information transfer in the parietal and occipital lobe (decreased Bc) and a compensated increase in the frontal lobe (decreased Nlp) were exhibited in both HIV+/cART+ and HIV+/cART– individuals (P < 0.05, FDR-corrected). Compared with HIV+/cART– and HCs, less specialized function segregation (decreased modularity and small-worldness property) and stronger integration in the network (increased Eglob and little changed path length) were found in HIV+/cART+ group (P < 0.05, FDR-corrected).

**Conclusion:**

Early HIV+ individuals exhibited a decrease in the efficiency of information transmission in sensory regions and a compensatory increase in the frontal lobe. HIV+/cART+ showed a less specialized regional segregation function, but a stronger global integration function in the network.

## Introduction

The human immunodeficiency virus (HIV) can cross the blood–brain barrier rapidly after seroconversion and then cause cognitive, behavioral, and motor abnormalities over time, that is, HIV-associated neurocognitive disorders (HAND) (1). HAND is divided into asymptomatic neurocognitive impairment (ANI), mild neurocognitive impairment (MND), and HIV-associated dementia (HAD). Combined antiretroviral therapy (cART) effectively reduced the incidence of HAD, but it does not completely prevent the development and progression of HAND. The prevalence of HAND is still 15–55% in HIV-infected patients with cART (HIV+/cART+) (2). The incidence varies according to the differences in age and duration of infection (3, 4). In the cART era, the milder forms of HAND account for the majority, but the cognitive impairment will still slowly aggravate (5), which may affect the performance of complex daily tasks and the compliance of cART (6). Although knowledge about HAND has increased in recent years, the latent neurobiological changes and mechanisms remain indefinite.

Different functional and structural magnetic resonance imaging (MRI) modalities have been extensively utilized in the studies of HAND and play a key role in early diagnosis, stage, prognosis, and neuropathological changes. Resting-state functional MRI (fMRI) demonstrated functional connectivity in cortico-striatal networks attenuated in treatment-naïve HIV individuals (HIV+/cART–), and HIV+/cART+ had functional connectivity strength similar to healthy controls (HCs) (7). The reduction was observed in the functional connectivity within the visual network (8), resting cerebral blood flow in the visual cortex and lenticular nuclei (9), and the amplitude of low-frequency fluctuations in the occipital lobe (10). Besides function, cerebral microstructures studies have shown significant abnormalities. The preferential impairments were observed in periventricular white matter (corpus callosum and corona radiata) based on diffusion tensor imaging (DTI) (11–13). In addition, the axonal chronic injury rather than demyelination in early HIV infection was proposed to be the possible pathogenesis of the neurological damage of HAND (12, 13). For gray matter, significant volumetric alterations were found in the subcortical region (thalamus, putamen, and caudate), parietal lobe, and posterior cortex in cognitively intact and milder forms of HAND assessed by high-resolution three-dimensional T_1_-weighted images (3D-T_1_WI) (8, 14–17). Moreover, the dynamic alterations of brain volume among subgroups of different cognitive stages of preclinical HAND were identified (17). In previous studies, several regional impairments that possibly underlie HAND have been found, which can provide a limited window to understand the whole network level of abnormalities in HAND.

The human brain is structurally organized into a complicated network, which is conducive to the effective processing and integration of information. Graph-based network topology analysis provides a powerful and non-invasive method to probe the topological properties of the whole brain and can help to reveal the underlying neuropathological mechanism. Research on the topological organization of white matter based on graph theoretic approaches revealed HIV+/cART^−^ had a lower clustering coefficient, weaker structural segregation, integration, and connection strength relative to HCs (18). Another study showed HIV+/cART+ had a lower global clustering coefficient indicative of brain network segregation and a lower nodal degree in the left thalamus compared with HCs (19).

Structural MRI is relatively insensitive to head motion and mental activity when compared with fMRI and DTI (20). In addition, although the structural networks based on DTI tractography represent the direct anatomical connection mode among brain regions, its effectiveness in mapping cortical networks is limited due to the highly isotropic water diffusion near cortical regions. An important hypothesis of structural covariance networks (SCNs) is that the morphological features of interregional gray matter are covariant because they have common effects on development, maturation, and disease transmission (20, 21). The SCN analysis based on morphological indices such as the gray matter volume and cortical thickness is considered to correspond with anatomical connectivity and can reflect the accurate coordination of cortical morphology (22, 23). Thus, using brain gray matter anatomy to investigate structural networks in cognitive disorders may be more stable and convincing to some degree (24) and has been widely applied in a variety of neuropsychiatric diseases nowadays (23, 25–28).

Cortical thickness can provide information on developmental maturity and genetics (29, 30), and relative to cortical volume, cortical thickness is more promising to reveal the underlying genetic architecture of brain structures (31). Another study explored the large-scale structural connection mode of the cerebral cortex by employing cortical thickness and found that the brain anatomical network had a strong small-worldness (32). These findings indicate that using cortical thickness to construct SCNs is an appropriate and more sensitive tool to investigate neuropsychiatric disorders. There has been one study on gray matter volume SCNs in HIV+ adolescents and found node properties disrupted and the hub distribution shifted (25). To date, the topological organization characteristics of SCNs in adult HIV+ individuals with or without cART are not clear. In this study, we explored the organization differences in cortical thickness SCNs in early HIV+ adults and investigated the effect of cART on network topology.

## Materials and methods

### Participants

A total of 65 right-handed HIV+ individuals (34 cases receiving stable cART for at least 6 months, HIV+/cART+; 31 drug-naïve cases, HIV+/cART–) and 35 matched HCs were collected from the infectious disease outpatient clinic of Beijing Youan Hospital. Demographic and clinical laboratory tests (age, gender, education, time since HIV diagnosis, time of infection without cART, time on cART, current plasma CD4+ cell count and CD4+/CD8+ ratio, and plasma viral load) were obtained from the patient's self-report and electronic medical records. All of the HIV+/cART+ were treated with tenofovir + lamivudine + efavirenz in this study. This study protocol was approved by the ethics committee of Beijing Youan Hospital, Capital Medical University, and was performed according to the criteria set by the Declaration of Helsinki. All participants signed written informed consent.

The inclusion criteria for HIV+ individuals were as follows: (1) Considering the maturation of brain anatomy (33), the age range of subjects was 25 to 50 years; (2) HIV+/cART+ had received stable cART for at least 6 months with undetectable plasma viral load; (3) HIV+/cART– did not experience any sort of cART. Inclusion criteria for HCs were healthy individuals between 25 and 50 years of age. The following exclusion criteria were applied to HIV and HCs groups: (1) a history of neurological or psychiatric diseases; (2) central nervous system infections and tumors; (3) brain trauma; (4) alcohol or drug abuse; (5) contraindications to MR.

### Neuropsychological tests

Within 1–3 h before MRI scan, to comprehensively assess the cognitive abilities, each HIV+ individual received a series of neuropsychological tests including a self-report of cognitive difficulties in daily life using a simple Activities of Daily Living scale questionnaire (34) and six cognitive domains. The cognitive domains and corresponding neurocognitive scales are listed in Table 1. The initial score for each test was converted to T-score and adjusted for gender, age, and education level. Averaged T-score was computed for the cognitive domain evaluated by various cognitive tests. If the performance is more than one standard deviation and < 2 standard deviations below the mean for a particular cognitive domain without decreased daily functioning, a diagnosis of ANI should be considered (35). Of the 65 HIV subjects, 26 were diagnosed with ANI and 39 were non-HAND. The differences in the cognitive abilities between HIV+/cART+ and HIV+/cART– are listed in Table 2.

**Table 1:** Cognitive domains and corresponding neurocognitive scales.

**Cognitive domains**	**Neurocognitive scales**
Verbal fluency	Animal verbal fluency test (AFT)
Executive function	Wisconsin card sorting tests (WCST-64)
Speed of information processing	Trail marking test A (TMT-A)
Fine motor skills	Grooved pegboard
Attention/working memory	①Continuous performance test-identical pair (CPT-IP) ②Wechsler memory scale (WMS-III) ③Paced auditory serial addition test (PASAT)
Learning and delayed recall	①Hopking verbal learning test (HVLT-R) ② Brief visuospatial memory test (BVMT-R)

**Table 2:** Demographic, clinical variables, and neuropsychological data.

	**HIV+/cART+** **(*n* = 34)**	**HIV+/cART–** **(*n* = 31)**	**HCs** **(*n* = 35)**	***p*-value**
Sex (M/F)	32/2	29/2	33/2	1.000^*†*^
Age (years), median (IQR)	29.5(25.75–36)	30(27–33)	33 (29–36)	0.273^*‡*^
Education level (years), median (IQR)	16(16–16.5)	16(16–16)	16(16–18)	0.825^*‡*^
Time since HIV diagnosis (months), median (IQR)	29.5(21–49.25)	4(3–11)	/	0.000^§,*^
Time of infection without cART, (months), median (IQR)	2(1–9)	4(3–11)	/	0.108^§^
Time on cART (months), median (IQR)	23.5(18–48)	/	/	/
CD4^+^ (cells/μl), median (IQR)	515(412.39–651.93)	431.85(346–570)	/	0.121^§^
CD4^+^/CD8^+^ ratio, median (IQR)	0.63(0.45–0.88)	0.45(0.33–0.54)	/	0.002^§,*^
Plasma viral load (IQR)	TND	4320(50–26533)	/	/
Scores of cognitive performance				
Verbal fluency (mean SD)	44.9 ± 8.9	46.1 ± 7.5	/	0.544^¶^
Attention/working memory, median (mean±SD)	40.9 ± 6.6	41.6 ± 6.1	/	0.648^¶^
Executive function, median (mean±SD)	55.7 ± 11.0	55.0 ± 7.8	/	0.783^¶^
Memory (learning/delayed recall) (mean±SD)	42.9 ± 8.6	43.3 ± 8.0	/	0.841^¶^
Speed of information processing (mean±SD)	45.0 ± 9.5	47.7 ± 9.4	/	0.257^¶^
Fine motor (mean±SD)	43.2 ± 9.4	47.3 ± 7.8	/	0.061^¶^
Cognitive status (ANI/non–HAND)	16/18	10/21	/	0.311^*†*^

HIV+/cART+, HIV individuals receiving stable cART; HIV+/cART–, therapy-naïve HIV individuals; HCs, healthy controls; M, male; F, female; IQR, interquartile range; TND, undetectable; ANI, asymptomatic neurocognitive impairment; HAND, HIV-associated neurocognitive disorders;

† χ^2^ test;

‡ Kruskal–Wallis test;

§ Mann–Whitney U-test;

¶ independent sample Student's t-test;

* significant level p < 0.05; /, no data available.

### Structural MRI acquisition

All participants underwent scanning using a 3.0 T Siemens MR scanner (Tim-Trio, Erlangen, Germany) with a 32-channel phased-array head coil at Beijing Youan Hospital. A latex head pad and earplugs were used to reduce head movement and scanner noise. First, T_2_-weighted fluid-attenuated inversion recovery combined fat saturation sequence [repetition time/echo time/inversion time (TR/TE/TI) = 8,000/97/2,370.9 ms] was obtained to exclude intracranial lesion. Subsequently, sagittal high-resolution 3D-T_1_WI were acquired using magnetization-prepared rapid gradient echo (MPRAGE). TR/TE/TI = 1,900/2.52/900 ms, field of view = 250 × 250 mm, acquisition matrix = 256 × 246, slice thickness = 1 mm, number of slices = 176, voxel size = 1 × 0.977 × 0.977 mm^3^, and flip angle = 9°.

### Image preprocessing

A simple visual assessment was first performed on the original T_1_-weighted images to ensure that there were no obvious motion artifacts, poor contrast, incomplete scanning, or brain atrophy. Subsequently, these images were format-converted and manually reoriented. All the T_1_-weighted images were then preprocessed using Computational Anatomy Toolbox (CAT12, http://www.neuro.uni-jena.de/cat/) within Statistical Parametric Mapping (SPM12, http://www.fil.ion.ucl.ac.uk/spm) based on MATLAB (R2016b, www.mathworks.com/). The converted images were entered into the preprocessing procedure in CAT12. The central surface reconstruction and cortical thickness measurement can be obtained in one step. Then, topology correction, spherical mapping, and spherical registration were carried out. The individual map of cortical thickness was smoothed by using a Gaussian filter with a full-width at half-maximum of 15 mm before statistical analysis (36).

### Construction of structural covariance network

Nodes and edges between nodes formed a network. The Desikan-Killiany Atlas with 68 parcels bilaterally (DK40 atlas) was usually employed in the construction of SCNs. According to DK40 Atlas, the nodes were defined by the classical cortical parcellation scheme (37), which was an automatic recognition scheme used to divide the cerebral cortex into 68 gyrus-based regions of interest. All subjects in each group (HIV+/cART+, HIV+/cART–, and HCs) were ordered by age (ascending sequence). The age-resolved structural networks were constructed using a sliding-window method (38, 39), which involved generating a series of overlapping “windows” (age bins) of subjects while incrementally sliding over the age range of the sample. Windows (age bins) were defined by equal sample size (window width) and incremental step length. The “window width” and “step length” (10 and 1, respectively, in the study) determined the number of windows. In each window, 68 brain regions correspond to 68 cortical thickness sequences (arranged in ascending order with age). Edges were defined by the inter-area Pearson correlation of cortical thickness sequence, which was obtained from each age bin. The strength of the edges was defined as Pearson's correlation coefficients. In each window width sequence, Pearson's correlation coefficients between the cortical thickness of all possible pairs of regions of interest were calculated to obtain a 68 × 68 Pearson correlation matrix, and then, each window (age bin) generated a structural correlation network. To obtain a series of 68 × 68 Pearson's correlation matrices for each group, regional cortical thickness values were cross-correlated within overlapping windows covering identical numbers of subjects and iteratively slid across the age range through regular increments. Last, the network parameters obtained from these matrices are compared among groups.

### Network analysis

GRETNA software was used for network analysis (40). The thresholds were set within the network density (sparsity) range. Sparsity was defined as the ratio of the number of edges in the network to the maximum possible number of edges. The lower bound of this range was determined as the minimum sparsity that all nodes in the network of each group were adequately connected (41). In the range of network sparsity 0.05–0.5 (42), with an interval of 0.05, a series of network parameters were calculated. This method normalized all generated networks to guarantee that group differences were not confounded by different numbers of nodes and edges due to the absolute threshold under a single sparsity and to ensure the thresholded networks were estimable for the small-worldness properties and the small-world index was 1.0. The area under the curve (AUC) was computed for each network index, providing a summary scalar for the topological characteristics of brain networks to prevent employing an arbitrary single threshold selection (43).

### Network metrics

Network metrics were computed by employing the graph theory network analysis (GRETNA) toolbox (40). Network metrics employed in this study contained the following: (1) regional nodal metrics: the mean nodal path length (Nlp) and betweenness centrality (Bc); (2) network community: the number of modules (mod_num) and the modularity of a network (Q); (3) large-scale metric: global efficiency (Eglob) and local efficiency (Eloc) at the network level; (4) small-world properties: normalized clustering coefficient (γ, Gamma), normalized characteristic path length (λ, Lambda), and small-worldness (σ, Sigma). The mathematical definitions, detailed interpretation, and clinical significance of the network metrics are listed in Table 3. The formulas and usages of each metric can also be found in the previous study (51, 52). The workflow employed in this study was shown in Supplementary Figure S1.

**Table 3:** Mathematical definitions, interpretation, and clinical significance of the network metrics (44).

**Network metrics, interpretation and clinical significance**	**Mathematical definitions**
**Basic concept and measures** **Basic concepts and notation**	*N* is the set of all nodes in the network, and *n* is the number of nodes. a_ij_ is the connection status between i and j.
**Degree:** number of links connected to a node	Degree of a node i (44) $k_{i}=\underset{j\in N}{\sum }a_{ij}$
**Number of triangles:** a basis for measuring segregation	Number of triangles around a node i (44) $t_{i}=\frac{1}{2}\underset{j,h\in N}{\sum }a_{ij}a_{ih}a_{jh}$
**Regional nodal metrics** **Shortest path length:** a basis for measuring integration. **Nlp** (the mean nodal path length) refers to the average of the shortest path length from a node to any other node in the network. The shorter Nlp, the faster the information transmission speed of the network.	The mean nodal path length (Nlp) of node i (45) $Nlp\;\left(i\right)=\;\frac{1}{N-1}\underset{i\neq j\in G}{\sum }dij$ Where N is the number of nodes in the netwotk G, and d_ij_ is the shortest path length between node i and j in the network.
**Bc:** the nodal betweenness for a given node characterizes its effect on information flow between other nodes. A node has a high Bc, which means that many or even all of the shortest paths between other nodes must pass through it. If this node disappears, the communication between other nodes will become difficult or even disconnected.	Betweenness centrality (Bc) of node *i* (46) $b_{i}=\frac{1}{\left(n-1\right)\left(n-2\right)}\;\underset{\begin{array}{c} h,j\in N\\ h\neq j,h\neq i,j\neq i \end{array}}{\sum }\frac{\rho_{hj}\left(i\right)}{\rho_{hj}}$ where *ρ_*hj*_* is the number of shortest paths between *h* and *j*, and *ρ_*hj*_* (*i*) is the number of shortest paths between *h* and *j* that pass through *i*.
**Network community** **Mod_num:** The module is a subset of tightly clustered nodes (subnetworks). The number of modules is the number of nodes clusters in a network under each threshold. **Modularity (Q):** a measure of segregation. A less modular network means fewer connections within modules and more connections with other modules.	Modularity of the network (47) $Q=\underset{u\in M}{\sum }\left[e_{uu}-{\left(\underset{v\in M}{\sum }e_{uv}\right)}^{2}\right]$ where the network is fully subdivided into a set of non-overlapping modules *M*, and *e*_uv_ is the proportion of all links that connect nodes in module *u* with nodes in module *v*.
**Large-scale metrics** **Eglob:** a measure of integration. Global efficiency measures the ability of parallel information transfer in a network. The higher the Eglob of the network, the faster the rate of information transmission between network nodes.	Global efficiency of the network (48) $E_{glob}=\frac{1}{n}\underset{i\in N}{\sum }E_{glob,i}=\frac{1}{n}\underset{i\in N}{\sum }\frac{\underset{j\in N,j\neq i}{\sum }d_{ij}^{-1}}{n-1}$ where *E*_i_ is the efficiency of node *i*.
**Eloc:** a measure of segregation. The local efficiency of the network measures how efficient communication is among the first neighbors of a given node when it is removed, which reflects the transmission capacity of local information of the network or how much the network is error tolerant.	Local efficiency of the network (48) $E_{loc}=\frac{1}{n}\underset{i\in N}{\sum }E_{loc,i}=\frac{1}{n}\underset{i\in N}{\sum }\frac{\underset{j,h\in N,j\neq i}{\sum }{a_{ij}a_{ih}\left[d_{jh}\left(N_{i}\right)\right]}^{-1}}{k_{i}\left(k_{i}-1\right)}$ where *E*loc, *i* is the local efficiency of node *i*, and _*d*j*h*_(*N*_i_) is the length of the shortest path between *j* and h, that contains only neighbors of *i*.
**Small-world properties:** The brain is not a completely random or regular network, but an ‘economic’ small-world network, it refers to a shorter characteristic path length than regular networks (high clustering and long path lengths) but greater local interconnectivity than random networks (low clustering coefficient and short path lengths). It supports both modularized and integrated information processing and maximizes the efficiency of information transfer at a relatively low wiring cost.	
**Gamma**, **γ:** The clustering coefficient (C) of a given node measures the likelihood its neighborhoods are connected, is considered as an index of information processing efficiency in local brain regions of brain network. **C:** a measure of segregation. The footmark real and random respectively represents the real and random networks to be analyzed.	Clustering coefficient of the network (49), $C=\frac{1}{n}\underset{i\in N}{\sum }C_{i}=\frac{1}{n}\underset{i\in N}{\sum }\frac{2t_{i}}{k_{i}\left(k_{i}-1\right)}$ where *C*_*i*_ is the clustering coefficient of node *i* (*C*_*i*_=0 for *k*_*i*_ < 2). Normalized clustering coefficient (γ) = Creal / Crandom.
**Lambda**, **λ:** The characteristic path length (L) is the average value of the shortest path between all node pairs, which reflects the overall efficiency of information integration between different brain regions. L: a measure of integration.	Characteristic path length of the network (49) $L=\frac{1}{n}\underset{i\in N}{\sum }L_{i}=\frac{1}{n}\underset{i\in N}{\sum }\frac{\underset{j\in N,j\neq i}{\sum }d_{ij}}{n-1}$ where *L*_*i*_ is the average distance between node *i* and all other nodes. Normalized characteristic path length (λ) = Lreal / Lrandom.
**Sigma**, **σ:** It reflects the balance between segregation and integration among all the nodes in the network.	Small-worldness (σ) = γ / λ. (50). The γ > 1, λ ≈ 1 and σ > 1 indicates that the network has small-worldness.

### Statistical analysis

#### Demographic and clinical variables

Demographic, clinical, and neuropsychological variables were analyzed utilizing SPSS 20.0 (IBM Inc. Armonk, NY, USA). First, all continuous variables were normally distributed and checked by using the Shapiro–Wilk test. For normally distributed variables, mean ± SD were present, and for non-normally distributed data, medians with interquartile range were present. Age and education level were not normally distributed, and the Kruskal–Wallis test was used to compare the differences among the three groups. The time since HIV diagnosis, time of infection without cART, CD4+ cell count, and CD4+/CD8+ ratio were not normally distributed, and the Mann–Whitney U-test was used to calculate the differences between the two patient groups, while neuropsychologic test scores were normally distributed, and independent two-sample *t*-test was used. The sex distribution among three groups and cognitive ability between two patient groups were evaluated by utilizing the chi-square test. All *p*-values <0.05 were considered statistically significant.

#### Network-based statistic

The one-way analysis of variance (ANOVA) and *post-hoc* tests employing network-based statistics (NBS) were performed to evaluate the group differences in the AUCs of all the network indices (53). The network metrics difference between groups was corrected for multiple comparisons by the false discovery rate (FDR) module of NBS with 5,000 permutations, and the significance threshold was set to *p* < 0.05.

## Results

### Demographic, clinical, and neuropsychological data

The baseline demographic, clinical, and neuropsychological data in different groups are shown in Table 2. No significant differences in gender, age, and education levels were observed among HIV+/cART+, HIV+/cART–, and HCs groups. No significant difference existed in the duration of HIV infection before therapy between the two HIV+ groups. Due to the need for antiviral treatment, HIV+/cART+ had a longer disease course. A higher CD4+/CD8+ ratio and a tendency for higher CD4+ levels in HIV+/cART+ were observed relative to HIV+/cART–, but the difference in CD4+ cell counts was not statistically significant. CD4+/CD8+ ratio is more stable, and the higher CD4+/CD8+ ratio implies immune recovery. Plasma viral load was controlled and undetectable in HIV+/cART+. It is important to note that most cognitive scale scores in HIV+/cART+ tended to be lower than those in HIV+/cART–, and about half of the patients in HIV+/cART+ were diagnosed with ANI. The results indicate cognitive impairment persists even with virus suppression and immune restoration on cART.

### Alterations in brain network properties

All the comparisons of brain network properties are shown in Table 4.

**Table 4:** *Post-hoc* analyses results of the altered network metrics among the three groups.

**Network metrics**	**HIV**+**/cART**+ **vs. HCs**		**HIV**+**/cART– vs. HCs**		**cART**+ **vs. cART–**	
	***t***	***p***	***t***	***p***	***t***	***p***
**Regional nodal metrics**						
**Nlp**						
SFG.L	−3.94857	0.00019	−4.23647	0.00008	NS	NS
SFG.R	−3.85112	0.00027	NS	NS	−4.27178	0.00007
**Bc**						
ITG.L	−4.75678	0.00001	NS	NS	NS	NS
LING.L	−5.54638	0.00000	−4.90126	0.00000	NS	NS
LING.R	NS	NS	NS	NS	−4.08712	0.00013
PCL.L	−7.15180	0.00000	−7.68687	0.00000	NS	NS
CAL.L	NS	NS	NS	NS	4.10505	0.00012
CAL.R	−5.33397	0.00000	NS	NS	−6.50684	0.00000
PoCG.L	−4.11746	0.00011	−4.61510	0.00002	NS	NS
PoCG.R	NS	NS	NS	NS	−4.21494	0.00008
PCUN.L	5.66704	0.00000	NS	NS	NS	NS
SPG.R	−8.27225	0.00000	NS	NS	−5.50156	0.00000
ACG.L	NS	NS	6.05058	0.00000	−7.09450	0.00000
**Network community**						
**Mod_num**						
0.20	2.49	0.01530	NS	NS	3.74	0.00041
0.25	NS	NS	NS	NS	4.15	0.00011
**Q**						
Q_0.05_	−3.83	0.00028	NS	NS	−3.79	0.00034
Q_0.10_	−3.86	0.00026	NS	NS	−5.53	0.00000
Q_0.15_	−3.91	0.00022	NS	NS	−5.53	0.00000
Q_0.20_	−4.14	0.00010	NS	NS	−5.79	0.00000
Q_0.25_	−5.03	0.00000	NS	NS	−6.22	0.00000
Q_0.30_	−4.94	0.00000	NS	NS	−5.67	0.00000
Q_0.35_	−5.18	0.00000	NS	NS	−5.46	0.00000
Q_0.40_	−5.61	0.00000	NS	NS	−5.85	0.00000
Q_0.45_	−5.33	0.00000	NS	NS	−6.07	0.00000
Q_0.50_	−5.95	0.00000	NS	NS	−6.61	0.00000
**Large-scale metrics**						
Eglob	6.55	0.00000	3.54	0.00075	3.99	0.00018
Eloc	5.34	0.00000	3.60	0.00063	2.47	0.01620
**Small-world properties**						
Gamma, γ	−4.48	0.00003	NS	NS	−4.13	0.00011
Lambda, λ	NS	NS	4.07	0.00013	−4.85	0.00000
Sigma, σ	−4.03	0.00014	NS	NS	−3.14	0.00259

HIV+/cART+, HIV individuals receiving stable cART; HIV+/cART–, therapy-naïve HIV individuals; HCs, healthy controls; Nlp, the average of the shortest-path length from a node to any other node in the network; SFG, superior frontal gyrus; L, left; R, right; NS, not significant; Bc, Betweenness centrality; ITG, inferior temporal gyrus; LING, lingual gyrus; PCL, paracentral lobule; CAL, pericalcarine cortex; PoCG, postcentral gyrus; PCUN, precuneus; SPG, superior parietal gyrus; ACG, anterior cingulate gyri; Mod_num, number of modules; Q, modularity; Eglob, global efficiency; Eloc, local efficiency; γ, normalized clustering coefficient; λ, normalized characteristic path length; σ, small-worldness. t-value, a positive value represents the network metric value of the former group, which is higher than the latter, and a negative value represents a decrease.

#### Between-group differences in regional nodal metrics

**Figure 1:**
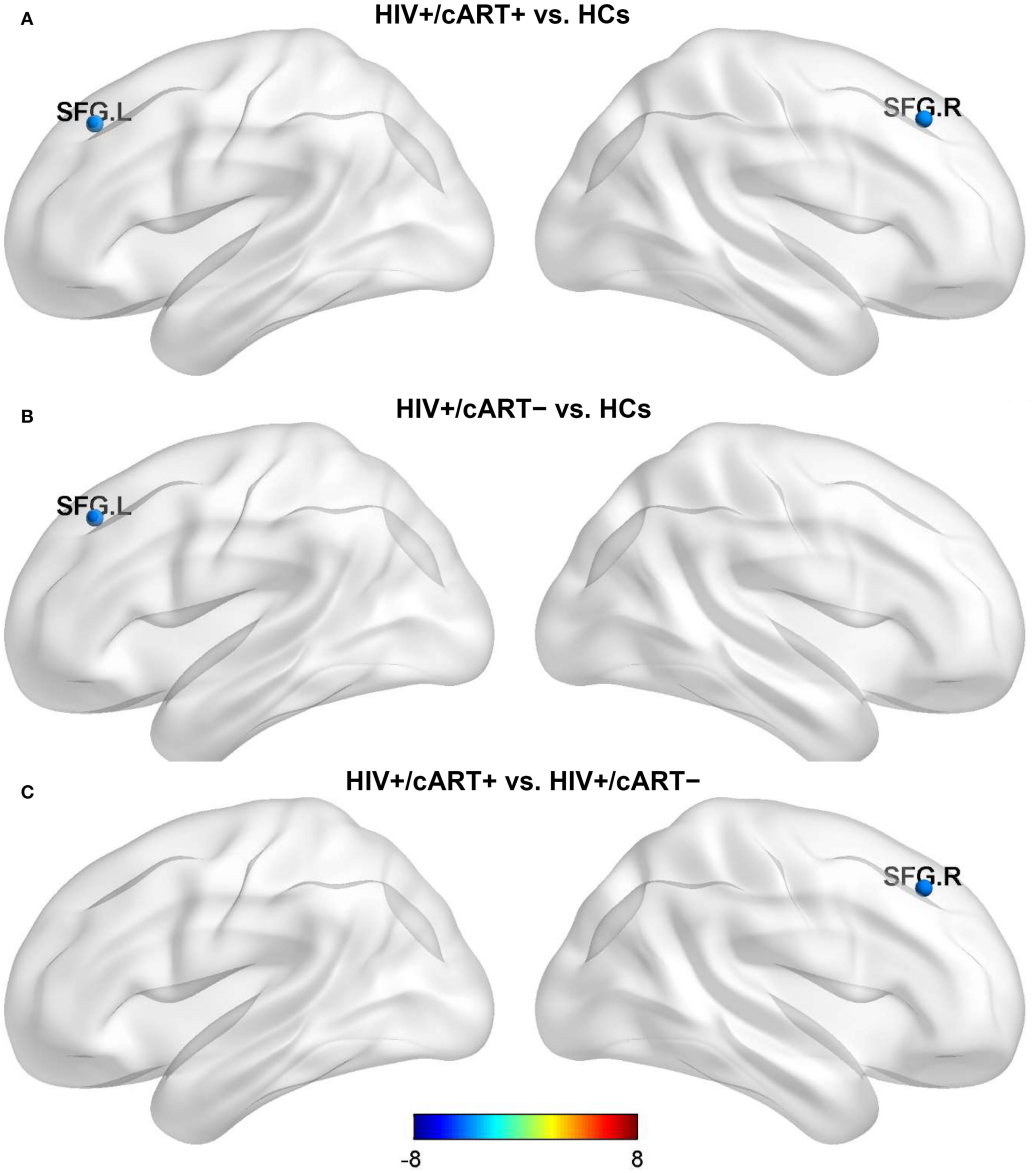
Between-group results in regional nodal metrics-Nlp. The color bar scales represent the t value of Nlp difference between groups, a positive t value represents the Nlp of the former group is higher than the latter, and a negative value represents a decrease (presented in blue). **(A)** HIV+/cART+ < HCs. **(B)** HIV+/cART– < HCs. **(C)** HIV+/cART+ < HIV+/cART–. Nlp, the average of the shortest-path length from a node to any other node in the network; HCs, healthy controls; SFG, superior frontal gyrus; L, left; R, right.

Nlp in the bilateral superior frontal gyrus (SFG) of the HIV+/cART+ group and Nlp in the left SFG of the HIV+/cART– group were both decreased compared to the HCs group (Figures 1A,B). In addition, Nlp in the right SFG had exhibited a reduction in HIV+/cART+ than in HIV+/cART– (Figure 1C).

**Figure 2:**
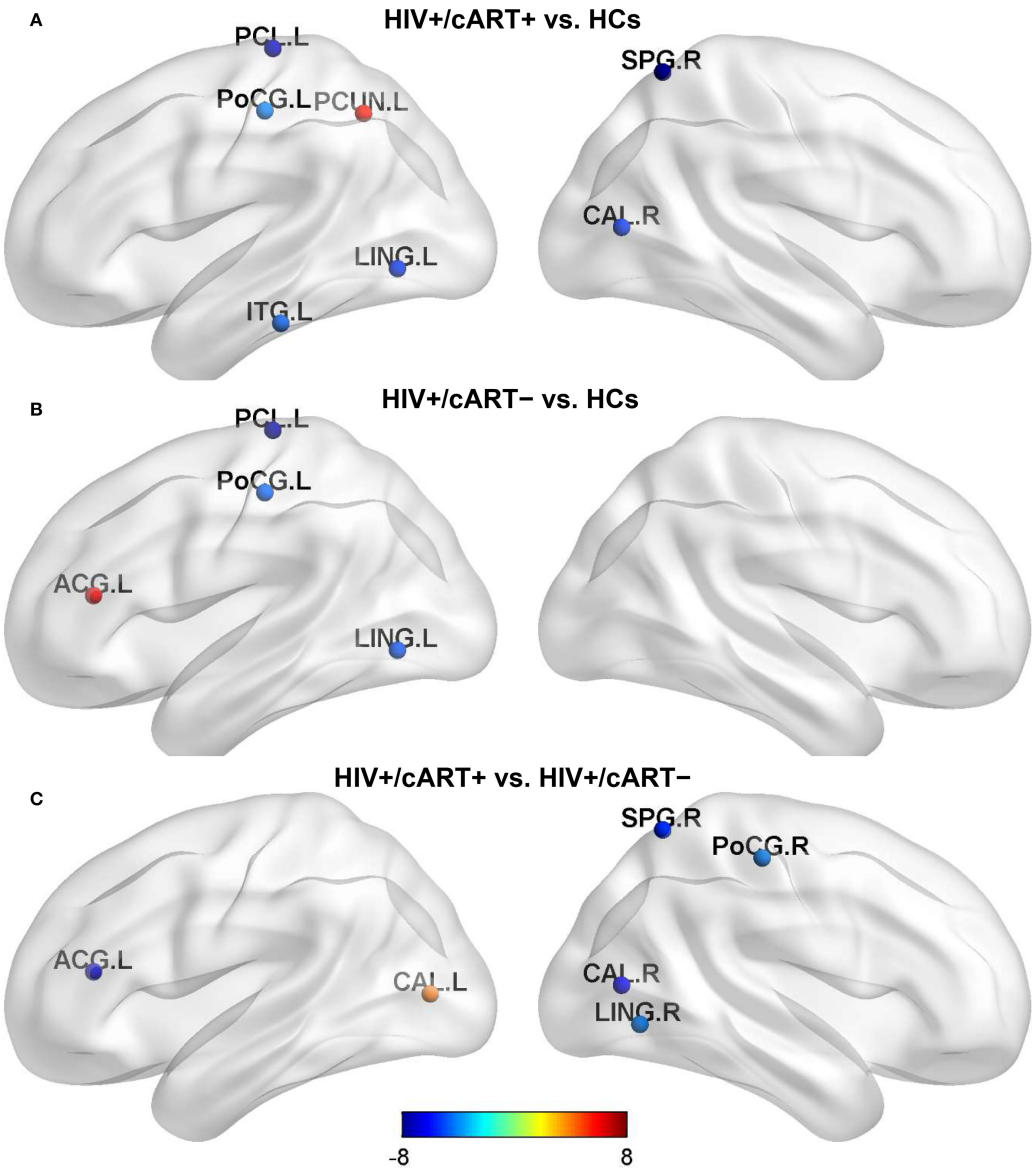
Between-group results in regional nodal metrics-Bc. The color bar scales represent the t value of Bc difference between groups. Blue (a negative t value) indicates that the former < the latter. Red and yellow (a positive t value) indicate that the former > the latter. Compared with HCs, Bc in the parietal lobe and occipital lobe decreased both in HIV+/cART+ **(A)** and HIV+/cART– group **(B)**, and the brain area of decreased Bc in HIV+/cART+ group was more extensive **(C)**. L, left; R, right; ITG, inferior temporal gyrus; LING, lingual gyrus; PCL, paracentral lobule; CAL, pericalcarine cortex; PoCG, postcentral gyrus; PCUN, precuneus; SPG, superior parietal gyrus; ACG, anterior cingulate gyri.

Compared with HCs, HIV+/cART+ showed increased Bc in the left precuneus (PCUN) and decreased Bc in the left paracentral lobule (PCL), postcentral gyrus (PoCG), lingual gyrus (LING), inferior temporal gyrus (ITG), the right superior parietal gyrus (SPG), and pericalcarine cortex (CAL) (Figure 2A); HIV+/cART– showed increased Bc in the left anterior cingulate gyri (ACG) and decreased Bc in the left PCL, PoCG, and LING (Figure 2B). Compared with HIV+/cART–, HIV+/cART+ showed increased Bc in the left CAL and decreased Bc in the left ACG, the right SPG, PoCG, CAL, and LING (Figure 2C).

#### Between-group differences in network community

**Figure 3:**
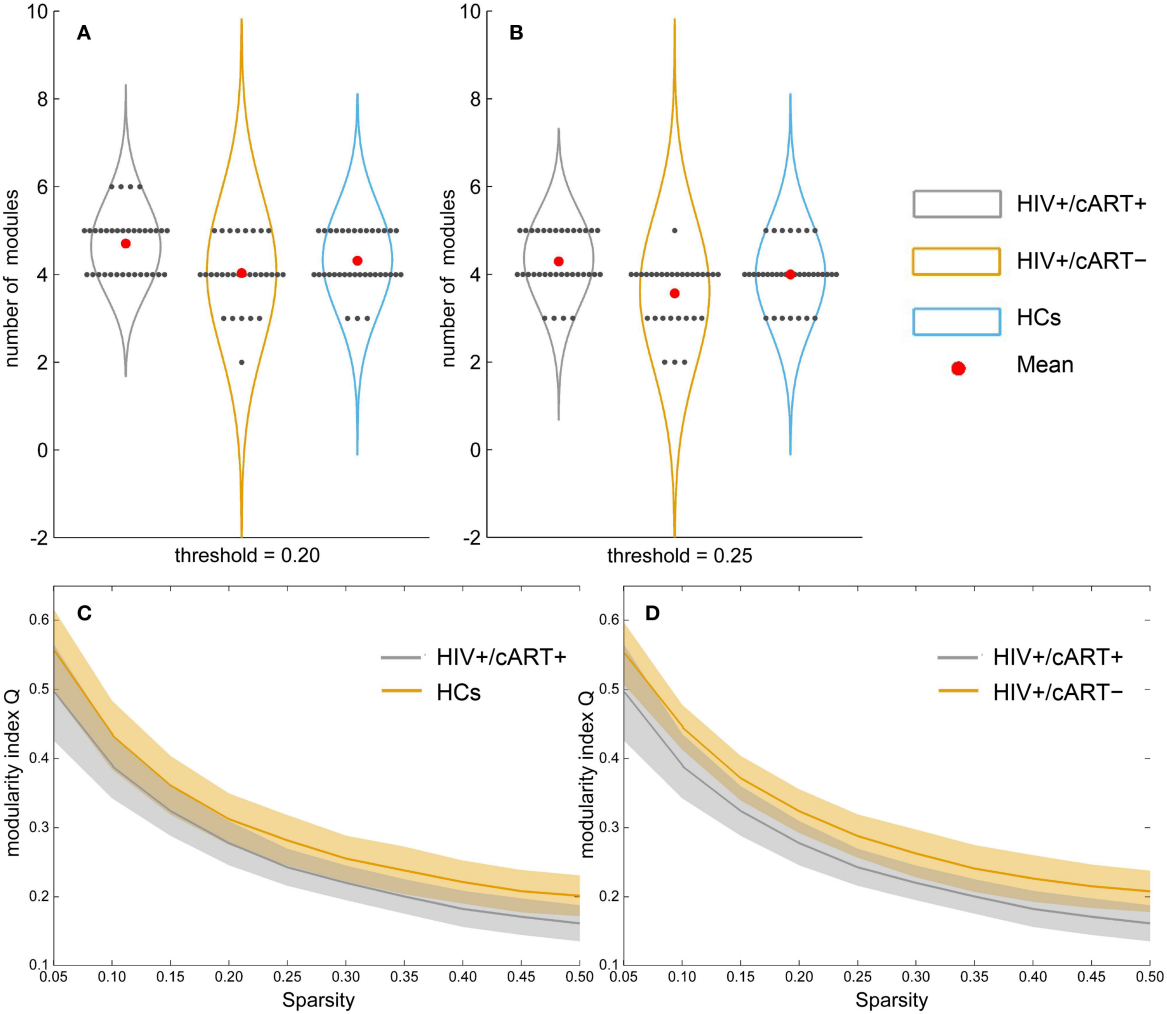
Between-group results in network community metrics number of modules **(A, B)** and modularity index Q **(C, D)**. At the threshold of 0.20, the number of modules in HIV+/cART+ was more than that in HCs (at the threshold of 0.20) and HIV+/cART– (at the threshold of 0.20 and 0.25). However, the modularity index Q of the HIV+/cART+ was significantly lower than that of the HCs and HIV+/cART– within the entire threshold range (0.05–0.50).

At the threshold of 0.20, the number of modules in HIV+/cART+ was more than that in HCs and HIV+/cART– (*p* < 0.05) (Figure 3A). At the threshold of 0.25, the number of modules in HIV+/cART+ was more than that in HIV+/cART- (*p* < 0.001) (Figure 3B). Under other thresholds, there was no difference in the number of modules among the three groups.

Within the entire threshold range (0.05–0.50), the modularity index Q of the HIV+/cART+ was significantly decreased than that of the HCs and HIV+/cART– (*p* < 0.001) (Figures 3C,D).

#### Between-group differences in large-scale metrics

**Figure 4:**
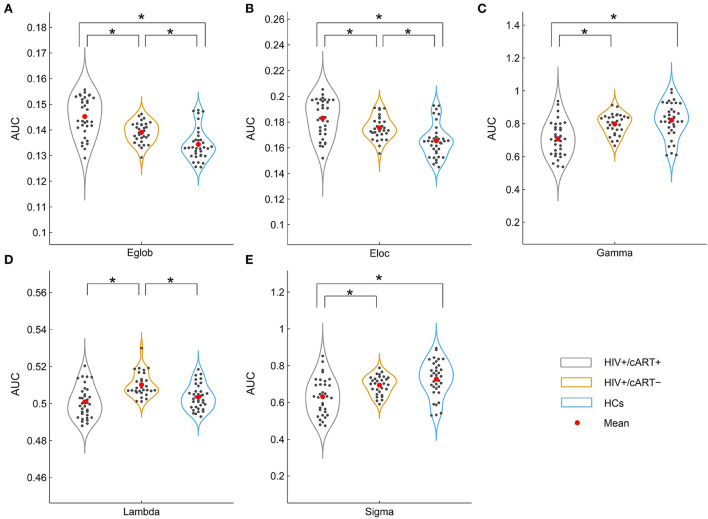
Between-group results in large-scale metrics **(A, B)** and small-world properties **(C–E)**. The asterisks designate significant differences between the two groups. AUC, area under the curve; Eglob, global efficiency; Eloc, local efficiency; HCs, healthy controls; γ, normalized clustering coefficient; λ, normalized characteristic path length; σ, small-worldness; *p value < 0.05.

The network efficiency analysis showed that the HIV+/cART+ and HIV+/cART– had greater Eglob and Eloc than HCs, and the HIV+/cART+ had larger Eglob and Eloc than the HIV+/cART– (Figures 4A,B).

#### Between-group differences in small-world properties

**Figure 5:**
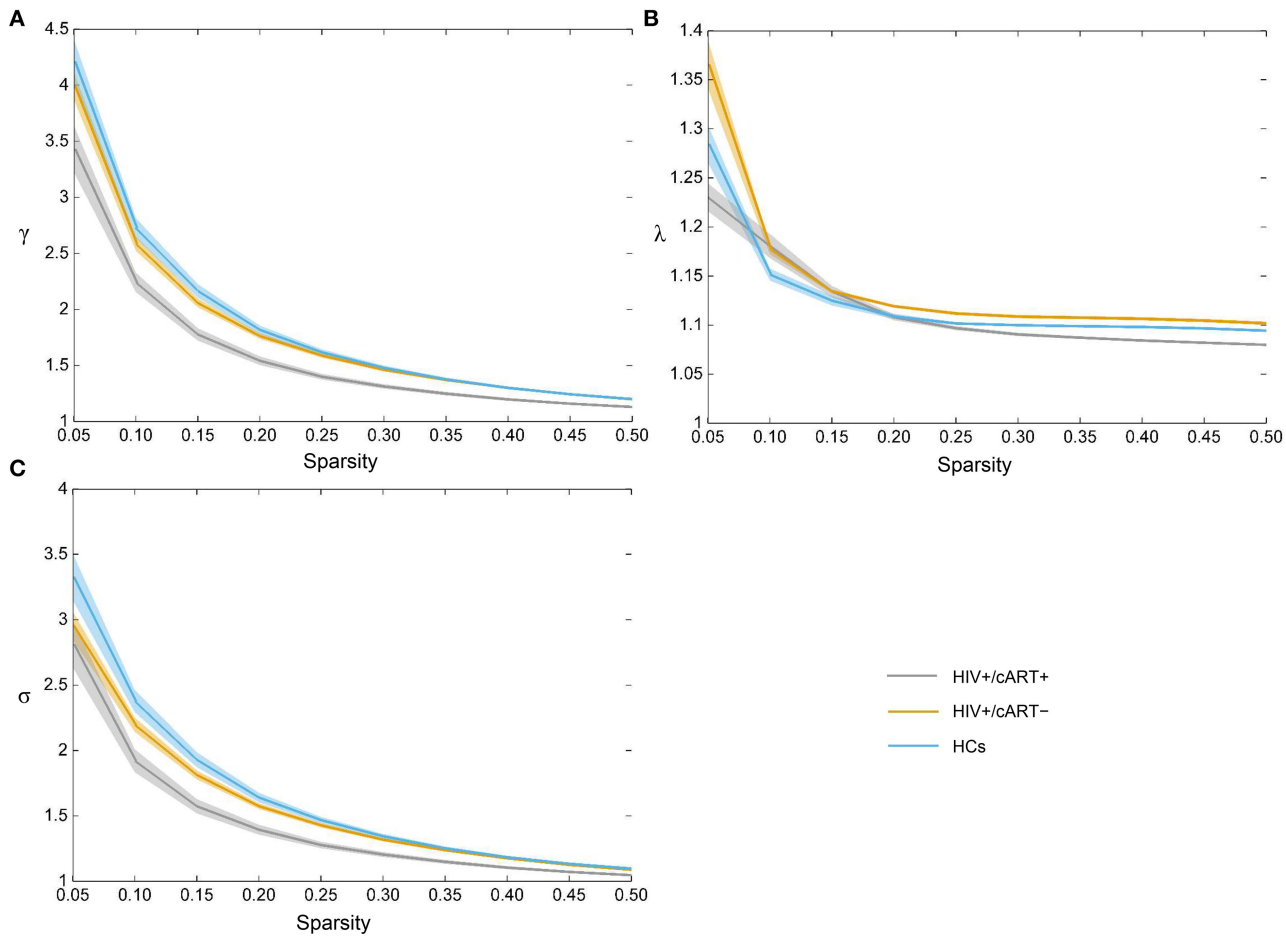
All three groups exhibited the typical features of small-worldness **(A)** γ > 1, **(B)** λ ≈ 1, and **(C)** σ > 1. γ, normalized clustering coefficient; λ, normalized characteristic path length; σ, small-worldness.

The γ > 1, λ ≈ 1, and σ > 1 indicated that the network exhibited a small-world topology. Despite the small-world topology that existed in the three groups (Figure 5), the small-worldness in HIV+/cART+ was lower than that in HIV+/cART– and HCs (Figures 4C–E).

## Discussion

To our knowledge, this is the first study to investigate the characteristics and differences of gray matter network topology constructed by using cortical thickness among HIV+/cART+, HIV+/cART–, and HCs. The basic structure of brain networks (node and connection) can be constructed from three spatial scales, namely, regional nodal level, network community, and large scale (54). From the nodal level point of view, both HIV+/cART+ and HIV+/cART– individuals exhibited more efficiency in terms of information transfer in the frontal lobe (decreased Nlp) and less efficiency in the sensory regions (decreased Bc). In addition, the brain regions with decreased Bc in HIV+/cART+ group were more extensive than HIV+/cART–. For network community, large scale, and small-worldness metrics, HIV+/cART+ showed less specialized segregation (decreased modularity, clustering, and small-worldness property). However, HIV+/cART+ exhibited a stronger integration in the network (increased Eglob and little changed path length). The present results provide new evidence for gray matter network alterations at different scales in HIV individuals with and without cART.

### Alterations of regional nodal level gray matter network metrics

The shorter Nlp and the higher Bc, the faster the information transmission efficiency of the network. Notably, HIV+/cART+ had decreased Nlp in the bilateral superior frontal gyrus (SFG) compared with HCs, whereas HIV+/cART– had decreased Nlp in the left SFG. In addition, a decreased Nlp in right SFG of HIV+/cART+ was observed. The SFG is located at the superior part of the prefrontal cortex and is implicated in a variety of functions, including motor, working memory, resting state, and cognitive control (55). Previous fMRI studies indicated that the alpha activity and spontaneous brain activity in the prefrontal cortex increased in HIV+/cART+ compared with HCs (10, 56). The effect of Nlp reduction in the SFG of HIV+ individuals on brain function is still unclear. It may be related to the reorganization of brain structure, and this organization structure has a positive impact on network efficiency and information transfer. It could also be a compensatory change, where the brain's compensatory measures are activated to increase the capacity of the frontal lobes to preserve cognitive performance.

The Bc in the left paracentral lobule (PCL), postcentral gyrus (PoCG), and lingual gyrus (LING) decreased in HIV+ individuals (both cART+ and cART–) compared to HCs. The PCL is the medial continuation of the precentral gyrus and postcentral gyrus, belonging to sensorimotor networks and responsible for the movement of the contralateral lower extremity and the control of micturition and defecation (57, 58). For HIV+ individuals, PCL involvement is relatively rare. Becker et al. reported that HIV+ individuals had abnormally reduced resting-state beta oscillations in PCL compared to HCs (59). The PoCG also belongs to sensorimotor networks, which is located in the parietal lobe of the cerebral cortex and is the site of the primary somatosensory cortex (57, 60). Moreover, the gray matter reductions are much more severe in the primary sensory regions of HIV+ individuals (61). As a part of medial occipital lobe, the lingual gyrus composes the visual networks with other tissues together and is necessary for basic and higher level visual processing (57, 62). Ances et al. have found reduced activation in occipital cortex during a visual stimulation task and decreased resting cerebral blood flow in the same visual regions using arterial spin labeling in HIV+ individuals relative to HCs (9, 63, 64). Decreased functional connectivity and spontaneous brain activity in occipital cortex have been reported in some studies (10, 65, 66). In addition to PCL, PoCG, and LING, Bc of inferior temporal gyrus (ITG), right superior parietal gyrus (SPG), and pericalcarine cortex (CAL) decreased in HIV+/cART+. ITG and CAL are also part of the visual networks (57). The reduction of Bc in HIV+ individuals presented in this study mainly involved the sensorimotor and visual networks, both of which belonged to the sensory cortical system. Moreover, a tendency for lower cognitive scale scores and more extensive brain regions related to Bc reduction was observed in the HIV+/cART+ group. There are three possible reasons. First, the disease course in HIV+/cART+ was longer than that of HIV+/cART– in this study. Previous studies have found that there is a tendency for brain damage progression due to continued neuroinflammation and glial activation along with the prolongation of HIV infection (67, 68). Second, many antiretroviral drugs did not penetrate the blood–brain barrier as effectively as HIV, and the brain had been considered to be a sanctuary for HIV causing chronic persistent damage (69, 70). Third, the patients in HIV+/cART+ group were all treated with free first-line therapeutic regimes (tenofovir + lamivudine + efavirenz). It is known that Efavirenz (EFV) is neurotoxic, and the CNS side effects of EFV include dizziness, insomnia, agitation, impaired concentration, amnesia, somnolence, dreaminess, and hallucinations (71, 72). In addition to direct damage to neurons, cART might also indirectly change brain function by affecting immune, glial, or endothelial cells (73).

Comprehensively considering the two metrics of microscale, we observed an interesting finding that is relative to HCs, the efficiency of information transmission decreased in parietal and occipital lobe (sensory regions) (Bc decreased), but increased in frontal lobe (Nlp decreased) in HIV+/cART+ and HIV+/cART– groups. Though some studies of HIV infection have demonstrated the activation differences in frontal lobe (HIV+ individuals > HCs) (74–78), other work has noted that the opposite pattern in sensory regions (HIV individuals < HCs) (9, 10, 61, 63–66). Studies that simultaneously captured the two patterns in the same group of HIV+ individuals were limited relatively. We supposed that this might be due to the gray matter SCNs using cortical thickness being sensitive to the detection of subtle neuropathology changes. A magnetoencephalography study showed significantly reduced theta responses in postcentral gyrus (sensorimotor networks) and increased alpha activity in prefrontal cortex of HIV+ individuals compared with HCs. Gray matter volume reduction was also found in postcentral gyrus (56). The results of present research indicate that the brain works as a whole by communicating in the network rather than separately.

### Between-group differences in network-level metrics

The module used in this study refers to the subnetworks. The research results showed that HIV+/cART+ had a higher number of modules, but lower the modularity index (Q) compared to HCs and HIV+/cART–. A lower Q means fewer connections within modules and more connections with other modules. The increase of interconnection between modules will lead to the rapid spread of disease pathological markers (neurodegenerative process) and the loss of specialization (51). The results indicated that these structural communities were spatially rearranged and the functional differentiation of HIV+/cART+ increased; however, the intramodule strength and regional connectedness were significantly reduced, and the quality of the functional differentiation region was not enough high.

The main characteristic of small-world network is economic, which could be explained by a short average shortest-path length and high clustering. The small-worldness (σ = γ / λ) supports both specialized/modularized and integrated/distributed information processing and maximizes the efficiency of information transfer at a relatively low wiring cost. In our research, although the brain networks of all three groups exhibited small-world characteristics, the small-worldness was significantly reduced in HIV+/cART+. In addition, relative to HCs, the increase of λ (path length) in HIV+/cART+ was not significant. Therefore, the reductions of σ were predominantly attributed to reductions of γ (clustering coefficient). A reduced clustering coefficient in HIV+/cART+ indicated a less specialized or modularized network organization. Compared with HIV+/cART–, all the γ, λ, and σ values in HIV+/cART+ decreased. The results indicated a less optimal balance between cost and efficiency in HIV+/cART+ individuals, and a lower clustering coefficient makes the networks closer to a random configuration. The retroviral drugs or disease progression with the extension of HIV infection time were a possible explanation for the lower small-worldness of HIV+/cART+.

Segregation (reflected by clustering and Eloc) and integration (reflected by path length and Eglob) are two main organizational principles of brain networks (79). Segregation refers to the regional clusters that interact to efficiently perform specific cognitive tasks, and integration refers to the interactions between separated clusters to achieve complex integrated tasks (79). An optimal balance between integration and segregation enables efficient information processing. Relative to HCs, though the Eloc in HIV+/cART+ increased, the clustering and modularity decreased. The lower clustering coefficient may reflect the dedifferentiation, which is a concept of insufficient structural connectivity, leading to the destruction of network segregation and thus the reduction of cognitive specialization. The results demonstrated that segregation and cognitive specialization in HIV+/cART+ might be deficient. Another study on the topological organization of white matter also showed HIV+/cART+ had a lower global clustering coefficient compared to HCs, indicative of deficient segregation in the brain structural network (19). The increased Eglob and little changed path length in HIV+/cART+ showed stronger integration in the network. In many networks, as in our results, the modularity and Eglob are inversely related, because a highly modular topology might require longer communication paths to integrate information in the network. The results suggest that there are more regional defects in HIV+/cART+, they are more likely to have abnormal alterations in regional segregation, and the segregation and integration are unbalanced. The mechanism of cART on brain network regional module separation and global integration is unknown. Abnormal structural network, including reduction of regional segregation, sub-optimized small-worldness, reduced network resilience to random, and strong global integration, probably is the neuroanatomical basis of HIV+/cART+. Further studies are necessary to clarify these initial findings.

With more effective cART, the life span of HIV-positive individual increases, making the management of HIV-associated cognitive impairment increasingly important. In addition, most cases of HIV-associated cognitive impairment are mild, and neurobiomarkers sensitive to early changes in network organization are critical for optimizing treatment. However, little is known about the whole brain network topology in HIV+ adults receiving and not receiving cART. This study revealed topological disorganization of brain gray matter in HIV+ individuals with plasma viral load suppression and no treatment history, including abnormalities in the efficiency of information transmission, structural segregation, and structural integration. Our findings provide strong evidence for function-related disruptions of network organization in HIV and extend previous work.

### Limitation

This study has several limitations. First, this was a cross-sectional study, and longitudinal and prospective designs in HIV+ individuals before and after cART are needed to better investigate the effect of cART on structural covariance networks. Second, as a nature of structural covariance network (SCN) analysis, they could only perform group-level comparisons so that the findings will not be used as a single-subject biomarker for patients. Thus, limited by the current image method, we did not evaluate the relationship between clinical factors, cognitive scale scores, and SCNs network metrics. Third, healthy controls did not undergo neuropsychological testing, and it was needed to include more detailed cognitive information of control subjects in future. Fourth, due to the rapid growth of men who have sex with men in China in recent years, most of the subjects in this study were male, which may prevent female HIV+ individuals benefit from this research. Fifth, though the DK40 atlas is usually employed in the construction of SCNs, the graph theory measures obtained from DK40 atlas may not be sufficiently robust (80). A further higher-resolution network needs to be constructed to obtain more robust graph theory measures.

## Conclusion

Our primary findings were that early HIV+ individuals exhibited a decrease in the efficiency of information transmission in parietal lobe and occipital lobe (sensory regions) and a compensatory increase in frontal lobe. HIV+/cART+ showed a less specialized regional segregation function, but a stronger global integration function in the network. This result provides new evidence for gray matter network alterations at different scales in early HIV+ individuals with and without cART.

## Data availability statement

The original contributions presented in the study are included in the article/Supplementary material, further inquiries can be directed to the corresponding author/s.

## Ethics statement

The studies involving human participants were reviewed and approved by the Ethics Committee of Beijing YouAn Hospital, Capital Medical University. The patients/participants provided their written informed consent to participate in this study.

## Author contributions

RL, YG, WW, ZJ, BR, GL, and HL involved in the design and conduct of this study. RL, YG, BR, and GL contributed to the data analysis, the results interpretation and wrote the first draft of the manuscript. RL, YG, BR, GL, and HL wrote portions of the manuscript and reviewed the whole manuscript. All authors contributed to the article and approved the submitted version.

## Funding

This work was supported by the National Key R&D Program of China [grant no. 2019YFE0121400]; National Natural Science Foundation of China [grant no. 82202118, 82271963, and 61936013]; Beijing Natural Science Foundation [grant no. 7212051]; Peking University Medicine Seed Fund for Interdisciplinary Research [grant no. BMU2018MX027]; Capital Medical University research and incubation funding [grant no. PYZ21129]; and Beijing Excellent Talent Plan [grant no. 2018000021469G290]. The funders and sponsors had no role in study design, data collection and analysis, decision to publish, or preparation of the manuscript.

## Conflict of interest

The authors declare that the research was conducted in the absence of any commercial or financial relationships that could be construed as a potential conflict of interest.

## Publisher's note

All claims expressed in this article are solely those of the authors and do not necessarily represent those of their affiliated organizations, or those of the publisher, the editors and the reviewers. Any product that may be evaluated in this article, or claim that may be made by its manufacturer, is not guaranteed or endorsed by the publisher.

## References

[1] Saylor D Dickens AM Sacktor N Haughey N Slusher B Pletnikov M. HIV-associated neurocognitive disorder - pathogenesis and prospects for treatment. Nat Rev Neurol. (2016) 12:309. 10.1038/nrneurol.2016.27 27080521 PMC5842923

[2] Heaton RK Clifford DB Franklin DR Woods SP Ake C Vaida F. HIV-associated neurocognitive disorders persist in the era of potent antiretroviral therapy: CHARTER Study. Neurology. (2010) 75:2087–96. 10.1212/WNL.0b013e318200d727 21135382 PMC2995535

[3] Makinson A Dubois J Eymard-Duvernay S Leclercq P Zaegel-Faucher O Bernard L. Increased prevalence of neurocognitive impairment in aging people living with human immunodeficiency virus: the ANRS EP58 HAND 55-70 study. Clin Infect Dis. (2020) 70:2641–8. 10.1093/cid/ciz670 31755936

[4] Cysique LA Casaletto KB Heaton RK. Reliably measuring cognitive change in the era of chronic HIV infection and chronic HIV-associated neurocognitive disorders. Curr Top Behav Neurosci. (2021) 50:271–98. 10.1007/978-3-030-80759-7 31559600

[5] Cysique LA Juge L Gates T Tobia M Moffat K Brew BJ. Covertly active and progressing neurochemical abnormalities in suppressed HIV infection. Neurol Neuroimmunol Neuroinflamm. (2018) 5:e430. 10.1212/NXI.0000000000000430 29312999 PMC5754644

[6] Letendre SL Ellis RJ Ances BM Mccutchan JA. Neurologic complications of HIV disease and their treatment. Top HIV Med. (2010) 18:45–55. 20516524 PMC5077300

[7] Ortega M Brier MR Ances BM. Effects of HIV and combination antiretroviral therapy on cortico-striatal functional connectivity. AIDS. (2015) 29:703–12. 10.1097/QAD.0000000000000611 25849834 PMC4391231

[8] Liu D Zhao C Wang W Wang Y Li R Sun J. Altered gray matter volume and functional connectivity in human immunodeficiency virus-infected adults. Front Neurosci. (2020) 14:601063. 10.3389/fnins.2020.601063 33343289 PMC7744568

[9] Ances BM Sisti D Vaida F Liang CL Leontiev O Perthen JE. Resting cerebral blood flow: a potential biomarker of the effects of HIV in the brain. Neurology. (2009) 73:702–8. 10.1212/WNL.0b013e3181b59a97 19720977 PMC2734291

[10] Li R Wang W Wang Y Peters S Zhang X Li H. Effects of early HIV infection and combination antiretroviral therapy on intrinsic brain activity: a cross-sectional resting-state fMRI study. Neuropsychiatr Dis Treat. (2019) 15:883–94. 10.2147/NDT.S195562 31114203 PMC6497505

[11] Tang Z Liu Z Li R Yang X Cui X Wang S. Identifying the white matter impairments among ART-naïve HIV patients: a multivariate pattern analysis of DTI data. Eur Radiol. (2017) 27:4153–62. 10.1007/s00330-017-4820-1 28396994

[12] Li RL Sun J Tang ZC Zhang JJ Li HJ. Axonal chronic injury in treatment-naive HIV+ adults with asymptomatic neurocognitive impairment and its relationship with clinical variables and cognitive status. BMC Neurol. (2018) 18:66. 10.1186/s12883-018-1069-5 29747571 PMC5943991

[13] Zhao T Chen J Fang H Fu D Su D Zhang W. Diffusion tensor magnetic resonance imaging of white matter integrity in patients with HIV-associated neurocognitive disorders. Ann Transl Med. (2020) 8:1314. 10.21037/atm-20-6342 33209894 PMC7661883

[14] Ragin B Du HY Ochs R Wu Y Sammet CL Shoukry A. Structural brain alterations can be detected early in HIV infection. Neurology. (2012) 79:2328–34. 10.1212/WNL.0b013e318278b5b4 23197750 PMC3578377

[15] Kallianpur KJ Jahanshad N Sailasuta N Benjapornpong K Chan P Pothisri M. Regional brain volumetric changes despite 2 years of treatment initiated during acute HIV infection. AIDS. (2020) 34:415–26. 10.1097/QAD.0000000000002436 31725432 PMC6994348

[16] Qi Y Ailixire A Gao YX Li RL Li HJ. Current situation and prospect of HIV-associated neurocognitive disorder research in China: epidemiology, research, diagnosis, and treatment status. AIDS Rev. (2021) 23:74–81. 10.24875/AIDSRev.20000044 33761523

[17] Li R Qi Y Shi L Wang W Zhang A Luo Y. Brain volumetric alterations in preclinical hiv-associated neurocognitive disorder using automatic brain quantification and segmentation tool. Front Neurosci. (2021) 15:713760. 10.3389/fnins.2021.713760 34456678 PMC8385127

[18] Baker LM Cooley SA Cabeen RP Laidlaw DH Joska JA Hoare J. Topological organization of whole-brain white matter in HIV infection. Brain Connect. (2017) 7:115–22. 10.1089/brain.2016.0457 28076974 PMC5359681

[19] Bell RP Barnes LL Towe SL Chen NK Song AW Meade CS. Structural connectome differences in HIV infection: brain network segregation associated with nadir CD4 cell count. J Neurovirol. (2018) 24:454–63. 10.1007/s13365-018-0634-4 29687404 PMC6105458

[20] Alexander-Bloch A Giedd JN Bullmore ET. Imaging structural co-variance between human brain regions. Nature Reviews Neuroscience. (2013) 14:322–36. 10.1038/nrn3465 23531697 PMC4043276

[21] Raznahan A Lerch JP Lee N Greenstein D Wallace GL Stockman M. Patterns of coordinated anatomical change in human cortical development: a longitudinal neuroimaging study of maturational coupling. Neuron. (2011) 72:873–84. 10.1016/j.neuron.2011.09.028 22153381 PMC4870892

[22] Hosseini SMH Mazaika P Mauras N Buckingham B Weinzimer SA Tsalikian E. Altered integration of structural covariance networks in young children with type 1 diabetes. Hum Brain Mapp. (2016) 37:4034–46. 10.1002/hbm.23293 27339089 PMC5053865

[23] Bruno JL Hosseini SMH Saggar M Quintin EM Raman MM Reiss AL. Altered brain network segregation in fragile X syndrome revealed by structural connectomics. Cerebral Cortex. (2017) 27:2249–59. 10.1093/cercor/bhw055 27009247 PMC5963822

[24] Zhang WJ Lei D Keedy SK Ivleva EI Eum S Yao L. Brain gray matter network organization in psychotic disorders. Neuropsychopharmacology. (2020) 45:666–74. 10.1038/s41386-019-0586-2 31812151 PMC7021697

[25] Li JL Gao L Wen Z Zhang J Wang PY Tu N. Structural covariance of gray matter volume in HIV vertically infected adolescents. Sci Rep. (2018) 8. 10.1038/s41598-018-19290-5 29352127 PMC5775353

[26] Liu H Jiang HX Bi WC Huang BS Li XJ Wang MM. Abnormal gray matter structural covariance networks in children with bilateral cerebral palsy. Front Hum Neurosci. (2019) 13. 10.3389/fnhum.2019.00343 31708758 PMC6819944

[27] Li YN Chu TP Che KL Dong FH Shi YH Ma H. Altered gray matter structural covariance networks in postpartum depression: a graph theoretical analysis. J Affect Disord. (2021) 293:159–67. 10.1016/j.jad.2021.05.018 34192630

[28] Zhao YJ Niu RN Lei D Shah C Xiao Y Zhang WJ. Aberrant gray matter networks in non-comorbid medication-naive patients with major depressive disorder and those with social anxiety disorder. Front Hum Neurosci. (2020) 14:172. 10.3389/fnhum.2020.00172 32587507 PMC7298146

[29] Docherty AR Sawyers CK Panizzon MS Neale MC Eyler LT Fennema-Notestine C. Research genetic network properties of the human cortex based on regional thickness and surface area measures. Front Hum Neurosci. (2015) 9. 10.3389/fnhum.2015.00440 26347632 PMC4542323

[30] Jha SC Xia K Schmitt JE Ahn M Girault JB Murphy VA. Genetic influences on neonatal cortical thickness and surface area. Hum Brain Mapp. (2018) 39:4998–5013. 10.1002/hbm.24340 30144223 PMC6218288

[31] Panizzon MS Fennema-Notestine C Eyler LT Jernigan TL Prom-Wormley E Neale M. Distinct genetic influences on cortical surface area and cortical thickness. Cereb Cortex. (2009) 19 2728–35. 10.1093/cercor/bhp026 19299253 PMC2758684

[32] He Y Chen ZJ Evans AC. Small-world anatomical networks in the human brain revealed by cortical thickness from MRI. Cerebral Cortex. (2007) 17:2407–19. 10.1093/cercor/bhl149 17204824

[33] Tamnes CK Østby Y Fjell AM Westlye LT Due-Tønnessen P Walhovd KB. Brain maturation in adolescence and young adulthood: regional age-related changes in cortical thickness and white matter volume and microstructure. Cereb Cortex. (2010) 20:534–48. 10.1093/cercor/bhp118 19520764

[34] Gandhi NS Skolasky RL Peters KB Moxley RTT Creighton J Roosa HV. A comparison of performance-based measures of function in HIV-associated neurocognitive disorders. J Neurovirol. (2011) 17:159–65. 10.1007/s13365-011-0023-8 21437751 PMC4022137

[35] Antinori A Arendt G Becker JT Brew BJ Byrd DA Cherner M. Updated research nosology for HIV-associated neurocognitive disorders. Neurology. (2007) 69:1789–99. 10.1212/01.WNL.0000287431.88658.8b 17914061 PMC4472366

[36] Fischl B Dale AM. Measuring the thickness of the human cerebral cortex from magnetic resonance images. Proc Natl Acad Sci U S A. (2000) 97:11050–5. 10.1073/pnas.200033797 10984517 PMC27146

[37] Destrieux C Fischl B Dale A Halgren E. Automatic parcellation of human cortical gyri and sulci using standard anatomical nomenclature. Neuroimage. (2010) 53:1–15. 10.1016/j.neuroimage.2010.06.010 20547229 PMC2937159

[38] Vasa F Seidlitz J Romero-Garcia R Whitaker KJ Rosenthal G Vertes PE. Adolescent tuning of association cortex in human structural brain networks. Cerebral Cortex. (2018) 28:281–94. 10.1093/cercor/bhx249 29088339 PMC5903415

[39] Vijayakumar N Ball G Seal ML Mundy L Whittle S Silk T. The development of structural covariance networks during the transition from childhood to adolescence. Sci Rep. (2021) 11:9451. 10.1038/s41598-021-88918-w 33947919 PMC8097025

[40] Wang JH Wang XD Xia MR Liao XH Evans A He Y. GRETNA: a graph theoretical network analysis toolbox for imaging connectomics. Front Hum Neurosci. (2015) 9:386. 10.3389/fnhum.2015.00386 26175682 PMC4485071

[41] Hosseini SM Hoeft F Kesler SR. GAT a graph-theoretical analysis toolbox for analyzing between-group differences in large-scale structural and functional brain networks. PLoS ONE. (2012) 7:e40709. 10.1371/journal.pone.0040709 22808240 PMC3396592

[42] Li H Yang J Yin L Zhang H Zhang F Chen Z. Alteration of single-subject gray matter networks in major depressed patients with suicidality. J Magn Reson Imaging. (2021) 54:215–24. 10.1002/jmri.27499 33382162

[43] Zhang J Wang J Wu Q Kuang W Huang X He Y. Disrupted brain connectivity networks in drug-naive, first-episode major depressive disorder. Biol Psychiatry. (2011) 70:334–42. 10.1016/j.biopsych.2011.05.018 21791259

[44] Rubinov M Sporns O. Complex network measures of brain connectivity: uses and interpretations. Neuroimage. (2010) 52:1059–69. 10.1016/j.neuroimage.2009.10.003 19819337

[45] Chen H Huang L Yang D Ye Q Guo M Qin R. Nodal global efficiency in front-parietal lobe mediated periventricular white matter hyperintensity (PWMH)-related cognitive impairment. Front Aging Neurosci. (2019) 11:347. 10.3389/fnagi.2019.00347 31920627 PMC6914700

[46] Freeman LC. Centrality in social networks: conceptual clarification. Soc Netw. (1978) 1:215–39. 10.1016/0378-8733(78)90021-7

[47] Newman ME. Fast algorithm for detecting community structure in networks. Phys Rev E Stat Nonlin Soft Matter Phys. (2004) 69:066133. 10.1103/PhysRevE.69.066133 15244693

[48] Latora V Marchiori M. Efficient behavior of small-world networks. Phys Rev Lett. (2001) 87:198701. 10.1103/PhysRevLett.87.198701 11690461

[49] Watts DJ Strogatz SH. Collective dynamics of ‘small-world’ networks. Nature. (1998) 393:440–2. 10.1038/30918 9623998

[50] Humphries MD Gurney K. Network ‘small-world-ness’: a quantitative method for determining canonical network equivalence. PLoS One. (2008) 3:e0002051. 10.1371/journal.pone.0002051 18446219 PMC2323569

[51] Salathé M Jones JH. Dynamics and control of diseases in networks with community structure. PLoS Comput Biol. (2010) 6:e1000736. 10.1371/journal.pcbi.1000736 20386735 PMC2851561

[52] Wang JH Zuo XN Gohel S Milham MP Biswal BB He Y. Graph theoretical analysis of functional brain networks: test-retest evaluation on short- and long-term resting-state functional MRI data. PLoS ONE. (2011) 6:e21976. 10.1371/journal.pone.0021976 21818285 PMC3139595

[53] Zalesky A Fornito A Bullmore ET. Network-based statistic: identifying differences in brain networks. Neuroimage. (2010) 53:1197–207. 10.1016/j.neuroimage.2010.06.041 20600983

[54] Sporns O Tononi G Kotter R. The human connectome: a structural description of the human brain. PLoS Comput Biol. (2005) 1:e42. 10.1371/journal.pcbi.0010042 16201007 PMC1239902

[55] Briggs RG Khan AB Chakraborty AR Abraham CJ Anderson CD Karas PJ. Anatomy and white matter connections of the superior frontal gyrus. Clin Anat. (2020) 33:823–32. 10.1002/ca.23523 31749198

[56] Wilson TW Heinrichs-Graham E Becker KM Aloi J Robertson KR Sandkovsky U. Multimodal neuroimaging evidence of alterations in cortical structure and function in HIV-infected older adults. Hum Brain Mapp. (2015) 36:897–910. 10.1002/hbm.22674 25376125 PMC4491915

[57] Allen EA Erhardt EB Damaraju E Gruner W Segall JM Silva RF. A baseline for the multivariate comparison of resting-state networks. Front Syst Neurosci. (2011) 5:2. 10.3389/fnsys.2011.00002 21442040 PMC3051178

[58] Patra A Kaur H Chaudhary P Asghar A Singal A. Morphology and morphometry of human paracentral lobule: an anatomical study with its application in neurosurgery. Asian J Neurosurg. (2021) 16:349–54. 10.4103/ajns.AJNS_505_20 34268163 PMC8244697

[59] Becker KM Heinrichs-Graham E Fox HS Robertson KR Sandkovsky U. Decreased MEG beta oscillations in HIV-infected older adults during the resting state. J Neurovirol. (2013) 19:586–94. 10.1007/s13365-013-0220-8 24297500 PMC3913174

[60] Zhou L Tian N Geng ZJ Wu BK Dong LY Wang MR. Diffusion tensor imaging study of brain precentral gyrus and postcentral gyrus during normal brain aging process. Brain Behav. (2020) 10:e01758. 10.1002/brb3.1758 32844600 PMC7559610

[61] Thompson PM Dutton RA Hayashi KM Toga AW Lopez OL Aizenstein HJ. Thinning of the cerebral cortex visualized in HIV/AIDS reflects CD4(+) T lymphocyte decline. Proc Natl Acad Sci U S A. (2005) 102:15647–52. 10.1073/pnas.0502548102 16227428 PMC1266080

[62] Palejwala AH Dadario NB Young IM O'Connor K Briggs RG Conner AK. Anatomy and White Matter Connections of the Lingual Gyrus and Cuneus. World Neurosurg. (2021) 151:E426–E37. 10.1016/j.wneu.2021.04.050 33894399

[63] Ances BM Vaida F Yeh MJ Liang CL Buxton RB Letendre S. HIV Infection and aging independently affect brain function as measured by functional magnetic resonance imaging. J Infect Dis. (2010) 201:336–40. 10.1086/649899 20047503 PMC2804778

[64] Ances B Vaida F Ellis R Buxton R. Test-retest stability of calibrated BOLD-fMRI in HIV- and HIV plus subjects. Neuroimage. (2011) 54:2156–62. 10.1016/j.neuroimage.2010.09.081 20932922 PMC3229916

[65] Wang X Foryt P Ochs R Chung JH Wu Y Parrish T. Abnormalities in resting-state functional connectivity in early human immunodeficiency virus infection. Brain Connect. (2011) 1:207–17. 10.1089/brain.2011.0016 22433049 PMC3621309

[66] Egbert AR Biswal B Karunakaran K Gohel S Pluta A Wolak T. Age and HIV effects on resting state of the brain in relationship to neurocognitive functioning. Behav Brain Res. (2018) 344:20–7. 10.1016/j.bbr.2018.02.007 29425918

[67] Gendelman HE Gelbard HA. Adjunctive and long-acting nanoformulated antiretroviral therapies for HIV-associated neurocognitive disorders. Curr Opin HIV AIDS. (2014) 9:585–90. 10.1097/COH.0000000000000111 25226025 PMC4231201

[68] Ferguson D Clarke S Berry N Almond N. Attenuated SIV causes persisting neuroinflammation in the absence of a chronic viral load and neurotoxic antiretroviral therapy. AIDS. (2016) 30:2439–48. 10.1097/QAD.0000000000001178 27258396 PMC5051525

[69] Whitehead N Potterton J Coovadia A. The neurodevelopment of HIV-infected infants on HAART compared to HIV-exposed but uninfected infants. AIDS Care. (2014) 26:497–504. 10.1080/09540121.2013.841828 24125015

[70] Ellero J Lubomski M Brew B. Interventions for neurocognitive dysfunction. Curr HIV/AIDS Rep. (2017) 14:8–16. 10.1007/s11904-017-0346-z 28110422

[71] Adkins JC Noble S. Efavirenz. Drugs. (1998) 56:1055–64. 10.2165/00003495-199856060-00014 9878993

[72] Decloedt EH Maartens G. Neuronal toxicity of efavirenz: a systematic review. Expert Opin Drug Saf. (2013) 12:841–6. 10.1517/14740338.2013.823396 23889591

[73] Nightingale S Winston A Letendre S Michael BD Mcarthur JC Khoo S. Controversies in HIV-associated neurocognitive disorders. Lancet Neurol. (2014) 13:1139–51. 10.1016/S1474-4422(14)70137-1 25316020 PMC4313542

[74] Chang L Speck O Miller EN Braun J Jovicich J Koch C. Neural correlates of attention and working memory deficits in HIV patients. Neurology. (2001) 57:1001–7. 10.1212/WNL.57.6.1001 11571324

[75] Ernst T Chang L Jovicich J Ames N Arnold S. Abnormal brain activation on functional MRI in cognitively asymptomatic HIV patients. Neurology. (2002) 59:1343–9. 10.1212/01.WNL.0000031811.45569.B0 12427881

[76] Chang L Tomasi D Yakupov R Lozar C Arnold S Caparelli E. Adaptation of the attention network in human immunodeficiency virus brain injury. Ann Neurol. (2004) 56:259–72. 10.1002/ana.20190 15293278

[77] Chang L Yakupov R Nakama H Stokes B Ernst T. Antiretroviral treatment is associated with increased attentional load-dependent brain activation in HIV patients. J Neuroimmune Pharmacol. (2008) 3:95–104. 10.1007/s11481-007-9092-0 18247124 PMC2745242

[78] Ernst T Yakupov R Nakama H Crocket G Cole M Watters M. Declined neural efficiency in cognitively stable human immunodeficiency virus patients. Ann Neurol. (2009) 65:316–25. 10.1002/ana.21594 19334060 PMC2734503

[79] Bullmore E Sporns O. The economy of brain network organization. Nat Rev Neurosci. (2012) 13:336–49. 10.1038/nrn3214 22498897

[80] Romero-Garcia R Atienza M Clemmensen LH Cantero JL. Effects of network resolution on topological properties of human neocortex. Neuroimage. (2012) 59:3522–32. 10.1016/j.neuroimage.2011.10.086 22094643

