# Supplementary figures and images for: Altered gray matter structural covariance networks in drug-naïve and treated early HIV-infected individuals
Source: Front Neurol. 2022 Sep 20;13:869871. doi: 10.3389/fneur.2022.869871 (PMC9530039; doi:10.3389/fneur.2022.869871)

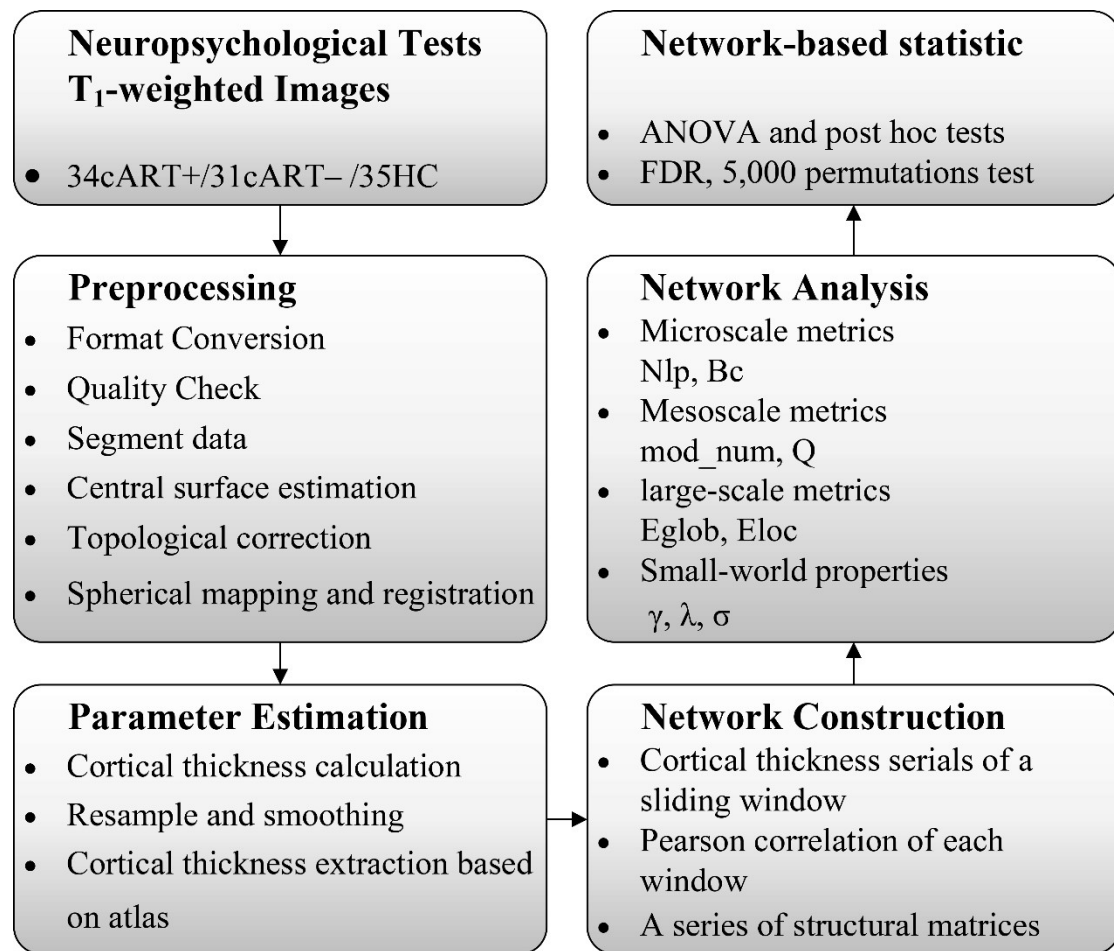

**FIGURE S1** Workflow of the image processing and analysis.

Supplement: Supplementary file 1 [file Image_1.PDF]
